# A Comprehensive Analysis of Common and Rare Variants to Identify Adiposity Loci in Hispanic Americans: The IRAS Family Study (IRASFS)

**DOI:** 10.1371/journal.pone.0134649

**Published:** 2015-11-24

**Authors:** Chuan Gao, Nan Wang, Xiuqing Guo, Julie T. Ziegler, Kent D. Taylor, Anny H. Xiang, Yang Hai, Steven J. Kridel, Jerry L. Nadler, Fouad Kandeel, Leslie J. Raffel, Yii-Der I. Chen, Jill M. Norris, Jerome I. Rotter, Richard M. Watanabe, Lynne E. Wagenknecht, Donald W. Bowden, Elizabeth K. Speliotes, Mark O. Goodarzi, Carl D. Langefeld, Nicholette D. Palmer

**Affiliations:** 1 Molecular Genetics and Genomics Program, Wake Forest School of Medicine, Winston-Salem, North Carolina, United States of America; 2 Center for Genomics and Personalized Medicine Research, Wake Forest School of Medicine, Winston-Salem, North Carolina, United States of America; 3 Center for Public Health Genomics, Wake Forest School of Medicine, Winston-Salem, North Carolina, United States of America; 4 Physiology and Biophysics, University of Southern California Keck School of Medicine, Los Angeles, California, United States of America; 5 Department of Preventive Medicine, University of Southern California Keck School of Medicine, Los Angeles, California, United States of America; 6 Institute for Translational Genomics and Population Sciences and Department of Pediatrics, Los Angeles Biomedical Research Institute at Harbor-UCLA Medical Center, Torrance, California, United States of America; 7 Department of Biostatistical Sciences, Wake Forest School of Medicine, Winston-Salem, North Carolina, United States of America; 8 Department of Research and Evaluation, Kaiser Permanente Southern California, Pasadena, California, United States of America; 9 Department of Cancer Biology, Wake Forest School of Medicine, Winston-Salem, North Carolina, United States of America; 10 Department of Internal Medicine, Strelitz Diabetes Center, Eastern Virginia Medical School, Norfolk, Virginia, United States of America; 11 Department of Diabetes and Metabolic Diseases Research, Beckman Research Institute of City of Hope, Duarte, California, United States of America; 12 Medical Genetics Research Institute, Cedars-Sinai Medical Center, Los Angeles, California, United States of America; 13 Department of Epidemiology, Colorado School of Public Health, University of Colorado Denver, Aurora, Colorado, United States of America; 14 Division of Public Health Sciences, Wake Forest School of Medicine, Winston-Salem, North Carolina, United States of America; 15 Center for Diabetes Research, Wake Forest School of Medicine, Winston-Salem, North Carolina, United States of America; 16 Department of Biochemistry, Wake Forest School of Medicine, Winston-Salem, North Carolina, United States of America; 17 Department of Internal Medicine, Division of Gastroenterology and Department of Computational Medicine and Bioinformatics, University of Michigan, Ann Arbor, Michigan, United States of America; 18 Division of Endocrinology, Diabetes, and Metabolism, Cedars-Sinai Medical Center, Los Angeles, California, United States of America; National Cancer Institute, National Institutes of Health, UNITED STATES

## Abstract

Obesity is growing epidemic affecting 35% of adults in the United States. Previous genome-wide association studies (GWAS) have identified numerous loci associated with obesity. However, the majority of studies have been completed in Caucasians focusing on total body measures of adiposity. Here we report the results from genome-wide and exome chip association studies focusing on total body measures of adiposity including body mass index (BMI), percent body fat (PBF) and measures of fat deposition including waist circumference (WAIST), waist-hip ratio (WHR), subcutaneous adipose tissue (SAT), and visceral adipose tissue (VAT) in Hispanic Americans (n_max_ = 1263) from the Insulin Resistance Atherosclerosis Family Study (IRASFS). Five SNPs from two novel loci attained genome-wide significance (P<5.00x10^-8^) in IRASFS. A missense SNP in the isocitrate dehydrogenase 1 gene (*IDH1)* was associated with WAIST (rs34218846, MAF = 6.8%, P_DOM_ = 1.62x10^-8^). This protein is postulated to play an important role in fat and cholesterol biosynthesis as demonstrated in cell and knock-out animal models. Four correlated intronic SNPs in the Zinc finger, GRF-type containing 1 gene (*ZGRF1*; SNP rs1471880, MAF = 48.1%, P_DOM_ = 1.00x10^-8^) were strongly associated with WHR. The exact biological function of *ZGRF1* and the connection with adiposity remains unclear. SNPs with p-values less than 5.00x10^-6^ from IRASFS were selected for replication. Meta-analysis was computed across seven independent Hispanic-American cohorts (n_max_ = 4156) and the strongest signal was rs1471880 (P_DOM_ = 8.38x10^-6^) in *ZGRF1* with WAIST. In conclusion, a genome-wide and exome chip association study was conducted that identified two novel loci (*IDH1 and ZGRF1*) associated with adiposity. While replication efforts were inconclusive, when taken together with the known biology, *IDH1* and *ZGRF1* warrant further evaluation.

## Introduction

Obesity is a global health problem closely associated with an increased risk for multiple metabolic diseases [1–3]. Body mass index (BMI) has been widely used in studies to estimate total body adiposity. However, BMI is derived from total body weight which possesses inter-individual variability attributed to muscle mass, i.e. BMI is not a direct measure of fat deposition, which is closely linked to health outcomes. Waist-hip ratio (WHR) and waist circumference (WAIST) have been well-recognized as complementary approaches to estimate fat deposition. However, they are often skewed by age and skeletal structure [4]. In addition to anthropometric measures, computed tomography (CT) has been recognized as the gold standard for measuring regional fat deposition [5]. Visceral adipose tissue (VAT) and subcutaneous adipose tissue (SAT) can be estimated by CT scans with both being strong risk factors for metabolic disturbances [6–8]. Alternatively, dual-energy X-ray absorptiometry (DEXA) can provide a direct measurement of total body fat volume [9] by partitioning total body mass into bone, lean, and fat soft tissue components.

Genome-wide association studies (GWAS) have been successful in identifying obesity-related loci with more than 100 loci identified to date [10–18]. However, over 80% of GWAS variants fall outside protein coding regions, which impairs causal inference [19]. In addition, associated variants possess small effect sizes providing limited information for disease risk prediction [20]. More recent evidence suggests low frequency and rare variants (minor allele frequency (MAF) <5%) also play a role in susceptibility to disease [21]. In addition, although the overall risk of obesity is much higher in Hispanic populations compared to non-Hispanic whites, i.e. 40.4% versus 34.3%, respectively [22], studies of the genetic contributors have been few in number and limited in scope in the Hispanic population. Until now, VIVA LA FAMILIA was the only cohort with published genome-wide significant obesity signals specific to the Hispanic population [23].

In this study, we hypothesized that genetic factors are responsible for the increased obese status in the Hispanic population. By combining more refined adiposity measures and genotypic information from GWAS and exome chip, we are able to conduct a comprehensive scan of the genome with the potential to identify ethnic specific causal variants.

## Materials and Methods

### Ethics Statement

Participants included in this study were recruited from clinical centers in San Antonio, TX and San Luis Valley, CO. The Institutional Review Board of each clinical (UT Health Science Center San Antonio Review Board and Colorado Multiple Institutional Review Board, respectively) and analysis (Wake Forest School of Medicine) site approved the study protocol and all participants provided their written informed consent.

### Study Participants

Study design and recruitment for the Insulin Resistance Atherosclerosis Family Study (IRASFS) have been described [24]. Briefly, the IRASFS was designed to identify the genetic and environmental basis of insulin resistance and adiposity. Hispanic Americans included in this report (n = 1417 individuals, 90 pedigrees) were recruited from clinical centers in San Antonio, TX and San Luis Valley, CO. While a diagnosis of diabetes was not required for participation, about 12.7% of genotyped individuals had diabetes. A detailed description of the phenotypes can be found in supplemental materials (S1 Text).

### Genotyping and Quality Control

GWAS genotyping was supported through the Genetics Underlying Diabetes in Hispanics (GUARDIAN) Consortium. Genotyping was attempted for 1039 Hispanic Americans plus 13 quality control (QC) duplicates using the Illumina OmniExpress Array (Illumina Inc.; San Diego, CA, USA; n = 730,525 markers) with an additional 14 external controls included to verify reproducibility across genotyping runs. Exome chip genotyping was carried out on the Illumina HumanExome Array v1.0 (n = 560) and v1.1 (n = 864) in the Center for Genomics and Personalized Medicine Research at Wake Forest School of Medicine, Winston-Salem, NC, USA. A detailed description of the quality control procedures can be found in supplemental materials (S1 Text). Overall, 687,094 polymorphic autosomal SNPs from the OmniExpress and 81,599 SNPs from the exome chip were analyzed in 1034 and 1263 individuals, respectively. Among them, 18,289 SNPs were overlapping between the two platforms. Genotype concordance rate was over 99.9%.

### Phenotypes

Anthropometric measures of adiposity were obtained using standard methods including height, weight, waist circumference (minimum between 10^th^ rib and iliac crest), and hip circumference (maximum circumference at the buttocks). BMI was calculated as weight in kilograms divided by height in meters squared. A CT scan was performed to estimate visceral and subcutaneous fat area (cm^2^). This procedure consisted of a single scout of the abdomen followed by a 10-mm thick axial image at the L4-L5 disc space using a standard protocol. CT images were read centrally at the University of Colorado Health Sciences Center. VAT and SAT were computed as previously described [25]. Percent body fat (PBF) was measured using DEXA at a 5 year follow-up exam, thus a reduced sample size as compared to other measures was available. A whole body DEXA scan uses the differential attenuation of two low dose x-ray beams to partition total body mass into bone, lean, and fat soft tissue components based on established mass-attenuation constants for bone mineral and lipid. Percent body fat (PBF) was calculated using total fat mass divided by measured weight x 100.

### Statistical Analysis for GWAS and Exome Chip

Phenotypes were transformed to best approximate the distributional assumptions of conditional normality and homogeneity of variance. Specifically, BMI, WAIST, and WHR were natural log transformed, SAT and VAT values were square-root transformed and PBF required no transformation. Admixture estimates were calculated using maximum likelihood estimation of individual ancestries using ADMIXTURE [26]. Specifically, the largest set of uncorrelated markers (r^2^<0.1) for K populations yielding the lowest cross validation (CV) error was used for unsupervised calculation of ancestral proportions. Representative ancestral populations from HapMap (CEU, YRI, CHN, and MEX) were included in the analysis. For GWAS, 117,347 LD-pruned SNPs for K = 5 populations (CV error = 0.48) were used. For exome chip, 10,566 uncorrelated SNPs for K = 5 populations (CV error = 0.52) were used. Three admixture estimates explained the largest amount of variation within the data and were highly correlated (r^2^>0.93) across platforms. Tests of association between individual variants and quantitative traits were computed using the Wald test from the variance component model implemented in Sequential Oligogenic Linkage Analysis Routines (SOLAR) [27]. Genetic models of association were calculated adjusting for age, gender, recruitment center, and admixture estimates. The primary inference was the additive genetic model. A lack of fit to the additive model was also tested using the orthogonal contrast (-1, 2,-1). If that lack of fit was significant (P<0.05), the model with the “best” p-value is the minimum of the dominant, additive, and recessive. Overall, the results were modestly inflated with inflation factors ranging from 1.04 to 1.08. QQ-plots of the six adiposity traits are shown in S1–S6 Figs. For robust estimation purposes, the additive and recessive genetic models were not computed if there were less than 10 and 20 individuals homozygous for the minor allele, respectively (similar to a minimal MAF of 1% and 2%). Conditional analysis was performed by adding the SNP with the strongest statistical significance to the model as a covariate.

### Power analysis

Power was computed using QUANTO (http://hydra.usc.edu/GxE).Simulations suggest that for these pedigrees the effective sample size equivalent to unrelated individuals for a quantitative trait is 92%. Thus, power calculations were based on a sample size of 951 for GWAS and 1162 for exome chip. The statistical power of our study to detect SNP-trait associations was computed assuming a type 1 error rate of α = 5.0x10^-8^. Overall, the OmniExpress had power of 0.70, 0.80, and 0.90 to detect SNP-trait associations that explain 3.7%, 4.1% and 4.7% of the trait variation, respectively. Similarly, the exome chip had power of 0.70, 0.80, and 0.90 to detect SNP-trait associations that explain 3.0%, 4.1% and 4.7% of the trait variation, respectively.

### De novo Genotyping in IRASFS and IRAS

In an effort to directly replicate the top association signals observed from exome chip and to search for potential causal SNPs at the *IDH1* and *ZGRF1* loci, a total of 76 SNPs were genotyped using the Sequenom MassARRAY Genotyping System (Sequenom, San Diego, CA, USA). Among these, 51 SNPs from the exome chip were chosen for genotyping in IRAS (n = 184) for replication (P<5.0x10^-5^). Another 25 SNPs (including 13 missense SNPs) within the *IDH1* and *ZGRF1* loci which were not covered by GWAS or exome chip were chosen for genotyping in IRASFS. Overall, genotyping efficiency was greater than 95%. To evaluate genotyping accuracy, 12 and 72 blind duplicate samples were included in IRAS and IRASFS, respectively. For all SNPs, genotyping was 99% concordant. PedCheck was computed for IRASFS genotype data and resulted in zeroing of 24 genotypes due to Mendelian inconsistencies [28]. Association analysis in IRASFS was computed using SOLAR as described. Analysis of data from IRAS was computed using QSNPGWA (https://www.phs.wakehealth.edu/public/home.cfm). Overall, 38 of the 51 SNPs were polymorphic and all SNP genotypes conformed to Hardy-Weinberg expectation (P>0.05).

### Replication and Meta-analysis

Six cohorts participating in the GUARDIAN consortium [29] provided in silico replication data: the Insulin Resistance Atherosclerosis Study (IRAS, n_max_ = 184) [30], BetaGene (n_max_ = 1218) [31–34], the Troglitazone in Prevention of Diabetes Study (TRIPOD, n_max_ = 125) [35, 36], the Hypertension-Insulin Resistance Family Study (HTN-IR n_max_ = 666) [37, 38], the Mexican-American Coronary Artery Disease Study (MACAD, n_max_ = 749) [39–41] and the NIDDM-Atherosclerosis Study (NIDDM-Athero, n_max_ = 179) [42]. A detailed description of the replication cohorts can be found in the supplemental materials (S1 Text). All cohorts, including IRASFS, were genotyped centrally as described above. All study protocols were approved by the local institutional review committees and all participants gave their informed consent.

A total of 71 GWAS SNPs (P<5.00x10^-6^) from the six adiposity phenotypes were selected for replication in the six cohorts in the GUARDIAN consortium. Meta-analysis of BMI, WAIST, and WHR was computed using the fixed effect model implemented in METAL (www.sph.umich.edu/csg/abecasis/metal/) as well as a random effect model in Metasoft [43] (http://genetics.cs.ucla.edu/meta/). For PBF, only IRASFS, BetaGene, MACAD, and HTN were included. For SAT and VAT, as they were not available in replication cohorts, a weighted meta-analysis of the p-values and samples sizes using surrogate phenotypes was performed. For example, BMI was used as the surrogate for PBF in IRAS, TRIPOD, and HTN-IR; BMI for SAT in all six replication cohorts; and WAIST for VAT in all six replication cohorts.

### Evaluation of previously identified signals

A total of 127 independent signals (r^2^<0.8) associated with adiposity and adiposity-related traits with genome-wide significance from previously published studies were evaluated [10]. A complete list of phenotypes used for the query can be found in supplemental material (S1 Text). Proxy SNPs (r^2^>0.8) for each of the 127 tag SNPs were also identified using SNAP Proxy Search [44] under the 1000 Genomes Pilot 1 SNP data set with a distance limit of 500kb. Association analysis was computed for all proxy SNPs with the six adiposity traits in IRASFS. Imputation of targeted variants not present on the OmniExpress Array was performed using IMPUTE2 [45]. All IRASFS samples genotyped on the OmniExpress Array (n = 1034) were imputed together using the 1000 Genomes Integrated Reference Panel (March 2012). In addition, 67 SNPs with associations to BMI and obesity from the 127 SNPs were selected for risk score analysis. The risk score was generated based on the number of risk alleles of the 67 SNPs. Associations of the risk score with six obesity phenotypes was conducted using SOLAR adjusting for age, gender, center, and admixtures.

## Results

Characteristics of the study samples are shown in Table 1. Across all studies there was a higher proportion of females. On average, individuals were overweight with a mean BMI greater than 28kg/m^2^. The IRASFS exome chip analysis included an additional 229 samples (n = 1263) compared to GWAS (n = 1034), of which 161 were individuals with T2D. This resulted in modestly increased means in adiposity-related traits.

**Table 1:** Demographic characteristics of the study populations.

	IRASFS		Replication Cohorts					
	GWAS	Exome Chip	IRAS	TRIPOD	BetaGene	HTN-IR	MACAD	NIDDM-Athero
**n**	1034	1263^c^	184	125	1218	666	749	179
**Male (%)**	41.1	41.1	41.1	0	28.4	40.6	43.3	41.8
**Age (years)** ^b^	40.6±13.7	42.8±14.6	54.0±8.2	34.8±6.3	34.5±8.2	37.4±14.2	34.5±8.8	31.8±9.69
**Body Mass Index (BMI; kg/m** ^**2**^)^b^	28.3±5.8	28.9±6.1	28.2±5.1	30.6±5.4	29.5±6.1	28.8±5.5	28.9±5.1	28.6±6.3
**Waist circumference (WAIST; cm)** ^b^	88.2±13.6	89.4±15.5	90.1±12.2	91.4±12.6	94.2±14.0	90.7±14.5	92.7±12.5	91.3±14.8
**Waist Hip Ratio (WHR)** ^b^	0.85±0.083	0.86±0.085	0.87±0.088	0.86±0.061	0.89±0.073	0.88±0.084	0.89±0.076	0.84±0.087
**Subcutaneous Adipose Tissue (SAT; cm** ^**2**^)^b^	329.8±151.7	339.1±154.7	NA	NA	NA	NA	NA	NA
**Visceral Adipose Tissue (VAT; cm** ^**2**^)^b^	106.6±57.0	114.0±61.3	NA	NA	NA	NA	NA	NA
**Percent Body Fat (PBF)** ^b^	33.5±8.7	34.0±8.7	NA	NA	34.4±8.4	32.6±9.1^a^	32.2±8.3	NA

^a^HTN-IR has only 139 individuals with PBF measurements

^b^Values are expressed as the mean ± standard deviation

^c^Includes 161 diabetics

Abbreviations: IRASFS, Insulin Resistance Atherosclerosis Family Study; IRAS, Insulin Resistance Atherosclerosis Study; TRIPOD, Troglitazone in Prevention of Diabetes Study; BetaGene, Family-based study of obesity, insulin resistance, and beta-cell dysfunction; HTN-IR, Hypertension-Insulin Resistance Family Study; MACAD, Mexican-American Coronary Artery Disease Study; NIDDM-Athero, NIDDM-Atherosclerosis Study.

**Fig 1:**
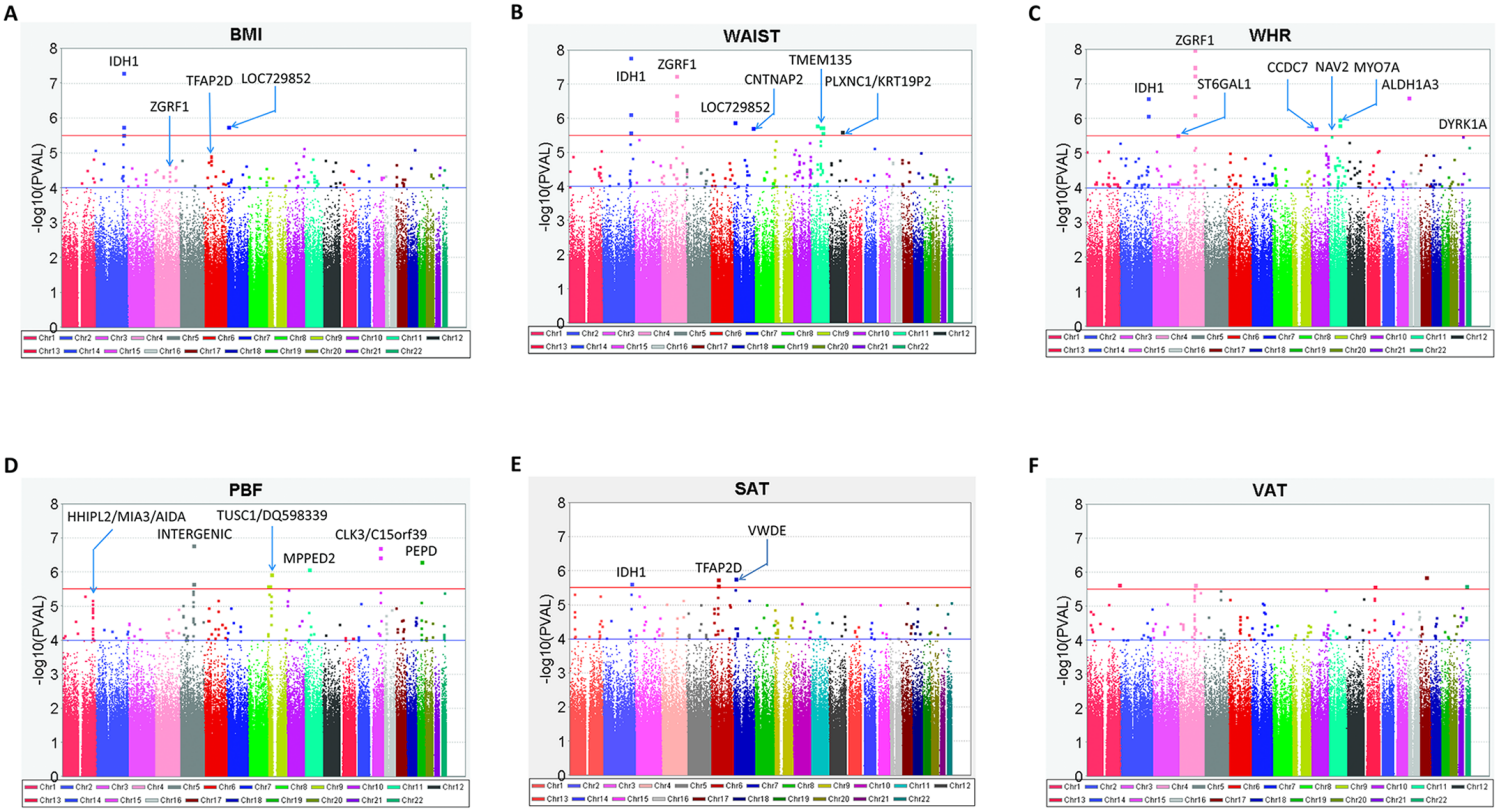
Manhattan plots for genome-wide and exome chip association analysis in IRASFS Hispanic Americans. (A). Body Mass Index (BMI), (B). Waist Circumference (WAIST), (C). Waist-Hip Ratio (WHR), (D). Subcutaneous Adipose Tissue (SAT), (E). Visceral Adipose Tissue (VAT), and (F). Percent Body Fat (PBF). Results were adjusted for age, gender, recruitment center (San Antonio, TX or San Luis Valley, CO), and admixture estimates. P-values are shown under the best fit model. The blue line at –log_10_(PVAL) = 4 represents a best P-value = 10^−4^ and the red line at –log_10_(PVAL) = 5.5 represents a best P-value = 3.16x10^-6^.

In IRASFS, 687,094 polymorphic autosomal SNPs from the OmniExpress and 81,599 SNPs from the exome chip were analyzed in 1034 and 1263 individuals, respectively. A summary of the association results are shown in Fig 1 and Table 2. In total, five SNPs from two loci reached genome-wide significance (P<5.00x10^-8^). Among these were four highly correlated SNPs (rs13144672, rs7696816, rs1471880, rs12054518; r^2^>0.96) associated with WHR in the Zinc finger, GRF-type containing 1 gene (*ZGRF1*). SNP rs1471880 (MAF = 48.1%), an intronic variant, showed the strongest signal of association under a dominant genetic model (WHR, P_DOM_ = 1.00x10^-8^) and explained 2.7% of the variance in WHR. On average, minor allele carriers have 2.3% lower of WHR (0.84±0.082 as compared to 0.86±0.085 in non-carriers). The second genome-wide significant signal was rs34218846 (MAF = 6.8%) with WAIST (P_DOM_ = 1.62x10^-8^). This SNP explains 2.1% of the phenotypic variance and marks a valine to isoleucine change (V178I) in the Isocitrate Dehydrogenase 1 gene (*IDH1*) on chromosome 2. De novo genotyping of additional, putatively functional SNPs at these loci in the IRASFS cohort did not identify additional statistically significant variants (S1 Table).

**Table 2:** Significant signals of association from genome-wide and exome chip association analyses in IRASFS.

SNP	Chr: Position (hg19)	Gene	Alleles^f^	N	MAF^e^	Beta+/-SE	P-Value
***Body Mass Index (BMI)***							
rs34218846^b^	2:209108317	*IDH1*	T/C	1253	0.070	-0.10±0.020	4.81E-08^c^
***Waist Circumference (WAIST)***							
rs34218846^b^	2:209108317	*IDH1*	T/C	1257	0.068	-0.080±0.010	1.62E-08^c^
***Waist-Hip Ratio (WHR)***							
rs1471880^a^	4:113546107	*ZGRF1*	C/A	1034	0.48	-0.027±0.0047	1.00E-08^c^
rs13144672^a^	4:113472958	*ZGRF1*	C/T	1034	0.47	0.027±0.0048	3.15E-08^d^
rs12054518^a^	4:113549989	*ZGRF1*	A/G	1034	0.48	0.027±0.0048	3.23E-08^d^
rs7696816^a^	4:113539969	*ZGRF1*	C/T	1034	0.48	0.027±0.0048	4.35E-08^d^

^a^SNP identified from GWAS

^b^SNP identified from exome chip

^c^Dominant Model

^d^Recessive Model

^e^Minor allele frequency based on the entire population

^f^Minor/Major allele on the positive strand

Replication of signals from the IRASFS GWAS (n = 71 SNPs with P<5.00x10^-6^) was attempted through meta-analysis with six additional Hispanic-American cohorts. Overall, no SNP attained genome-wide significance after meta-analysis (Table 3 and S2 and S3 Tables). The most significant signal remained to be rs1471880 (P_DOM_ = 8.38x10^-6^) at the *ZGRF1* locus associated with WAIST, which was also the strongest signal identified by GWAS (WHR, P_DOM_ = 1.00x10^-8^, WAIST P_DOM_ = 6.47x10^-7^). Among replication cohorts, similar allele frequencies and a consistent direction of effect were observed in five of the larger cohorts while the two smaller cohorts, TRIPOD (n = 125) and NIDDM-Athero (n = 179), had an opposite direction of effect (S7 Fig). For *IDH1*, the top SNP rs34218846 was identified from exome chip and was not available for in silico replication among the additional cohorts. Analysis of two GWAS proxy SNPs, rs6435435 (r^2^ = 0.91 with rs34218846, P_DOM_ = 1.73x10^-6^ for BMI) and rs6734788 (r^2^ = 0.37 with rs34218846, P_ADD_ = 7.33x10^-7^ for WAIST), near *IDH1* resulted in decreased significance (rs6435435 P_DOM_ = 0.11 with BMI and rs6734788 P_ADD_ = 7.98x10^-4^ with WAIST) with inconsistent directions of effect. *De novo* genotyping of variants at the *IDH1* locus in IRAS (n = 187) revealed five nominally associated SNPs (P<0.05), of which two SNPs, rs12105636 (BMI P_ADD_ = 0.046) and rs16840781 (BMI P_DOM_ = 0.030), were significant with a consistent direction of effect. However, the top *IDH1* missense SNP (rs34218846) was not significant (WAIST P_ADD_ = 0.45) with an opposite direction of effect (S4 Table). SNP rs12105636 and rs16840781 had nominal association signals in the IRASFS GWAS (WAIST P_DOM_ = 3.94x10^-3^ and P_DOM_ = 2.33x10^-3^, respectively) and were poorly correlated with rs34218846 (r^2^ = 0.34).

**Table 3:** Fixed-effect meta-analysis results (P<2.0x10^-3^) for significant signals of association (5.00x10^-6^) from IRASFS.

SNP	Chr:Position (hg19)	Alleles^b^	Effect	SE	Weight	Zscore	Direction^f^	*Gene Name*	P-value
***Waist Circumference (WAIST)*** ^a^									
rs1471880	4:113546107	A/C	0.0221	0.005			+++-++-	*ZGRF1*	8.38E-06^d^
rs6435435	2:209112551	A/G	0.0307	0.0077			+-++-++	*IDH1*	6.74E-05^d^
rs13144672	4:113472958	A/G	-0.0131	0.0033			---+--+	*ZGRF1*	6.90E-05^c^
rs7696816	4:113539969	A/G	-0.0126	0.0033			---+--+	*ZGRF1*	1.01E-04^c^
rs12054518	4:113549989	A/G	0.0125	0.0033			+++-++-	*ZGRF1*	1.24E-04^c^
rs1869479	11:44343856	A/C	0.0176	0.0047			+-+-++-	*HSD17B12/CD82*	2.01E-04^d^
rs6734788	2:209093069	A/G	-0.0194	0.0058			-+-++++	*CCNYL1/IDH1*	7.98E-04^c^
rs7937515	11:71841325	A/G	0.0141	0.0046			+-+-++-	*FAM86C1/FOLR3*	2.00E-03^c^
***Waist-Hip Ratio (WHR)*** ^a^									
rs7696816	4:113539969	A/G	-0.0116	0.0028			---++--	*ZGRF1*	4.34E-05^e^
rs13106629	4:113459416	A/G	0.0116	0.0028			+++--++	*C4orf32/ZGRF1*	4.39E-05^e^
rs12054518	4:113549989	A/G	0.0115	0.0028			+++--++	*ZGRF1*	5.43E-05^e^
rs2129405	4:113447137	A/G	0.0113	0.0028			+++--++	*C4orf32/ZGRF1*	6.24E-05^e^
rs10770244	12:17848331	A/G	-0.0091	0.0023			---++-+	*MIR3974/Y_RNA*	6.67E-05^d^
rs13144672	4:113472958	A/G	-0.0111	0.0028			---++--	*ZGRF1*	7.22E-05^e^
rs6734788	2:209093069	A/G	-0.0109	0.0028			-+-++-+	*CCNYL1/IDH1*	9.02E-05^c^

^a^Meta-analysis was computed based on beta and SE

^b^ Reference/alternate allele

^c^Additive model

^d^Dominant model

^e^Recessive model

^f^Direction follows as: IRASFS, IRAS, BetaGene, TRIPOD, HTN-IR, MACAD, NIDDM-Athero

In addition to the search for novel adiposity variants, 127 independent signals (r^2^<0.8) associated with adiposity and adiposity-related traits with genome-wide significance from previously published studies were evaluated in the IRASFS. Among these, 116 SNPs were directly genotyped or successfully imputed in IRASFS (S5 Table). Overall, 71 SNPs showed nominal association (P<0.05) with consistent direction of effect for at least one of the six adiposity traits. These included 23 SNPs for BMI, 17 SNPs for WAIST, 13 SNPs for WHR, 31 SNPs for SAT, 21 SNPs for VAT, and 13 SNPs for PBF. A two-sided nonparametric sign test was computed for the p-value thresholds of 0.10, 0.05, 0.01, and 4.31x10^-4^ (based on a Bonferroni correction of 116 variants) and the results were summarized in S6 Table. In brief, significantly higher replication signal concordance was observed with SAT and VAT (P<0.05). However, no replication signal survived Bonferroni correction. The strongest signal observed was rs2820464 located intergenically between lysophospholipase-like 1 gene (*LYPLAL1*) and solute carrier family 30, member 10 gene (*SLC30A10*) associated with SAT (P_ADD_ = 7.06x10^-4^). This variant was identified in a European cohort for an association with WHR (P = 7.00x10^-9^) [15]. Risk score analysis of the 67 previously identified obesity SNPs showed the strongest signal for SAT (P = 5.9x10^-4^). BMI, WAIST, and PBF were nominally associated with P-values 2.2x10^-3^, 7.7x10^-3^, and 3.2x10^-3^, respectively. Not surprising, VAT (P = 0.22) and WHR (P = 0.83) were not associated with the risk score as they are measures of adiposity depositions instead of total fat volumes.

## Discussion

Here we present a combined study of genome-wide and exome chip arrays to investigate the genetic determinants of adiposity measures in the Hispanic-American population. The complementary approach of using GWAS and exome chip enabled a broader coverage of both common and rare functional variants, resulting in an increased chance to identify causal mutations. Obesity-related traits evaluated included anthropometric (WAIST, WHR, and BMI), CT (SAT and VAT), and DEXA (PBF) measures. The assessment of CT and DEXA scans provided more accurate estimates of regional and total adiposity, respectively. We evaluated associations among Hispanic Americans from IRASFS (n_max_ = 1263) using GWAS and exome chip analysis with replication in six independent Hispanic cohorts (n_max_ = 4155). Association studies revealed *ZGRF1* and *IDH1* as two possible novel adiposity-related loci: *ZGRF1* was associated with waist-hip ratio (P_DOM_ = 1.00x10^-8^) and *IDH1* was associated with waist circumference (P_DOM_ = 1.62x10^-8^).

**Fig 2:**
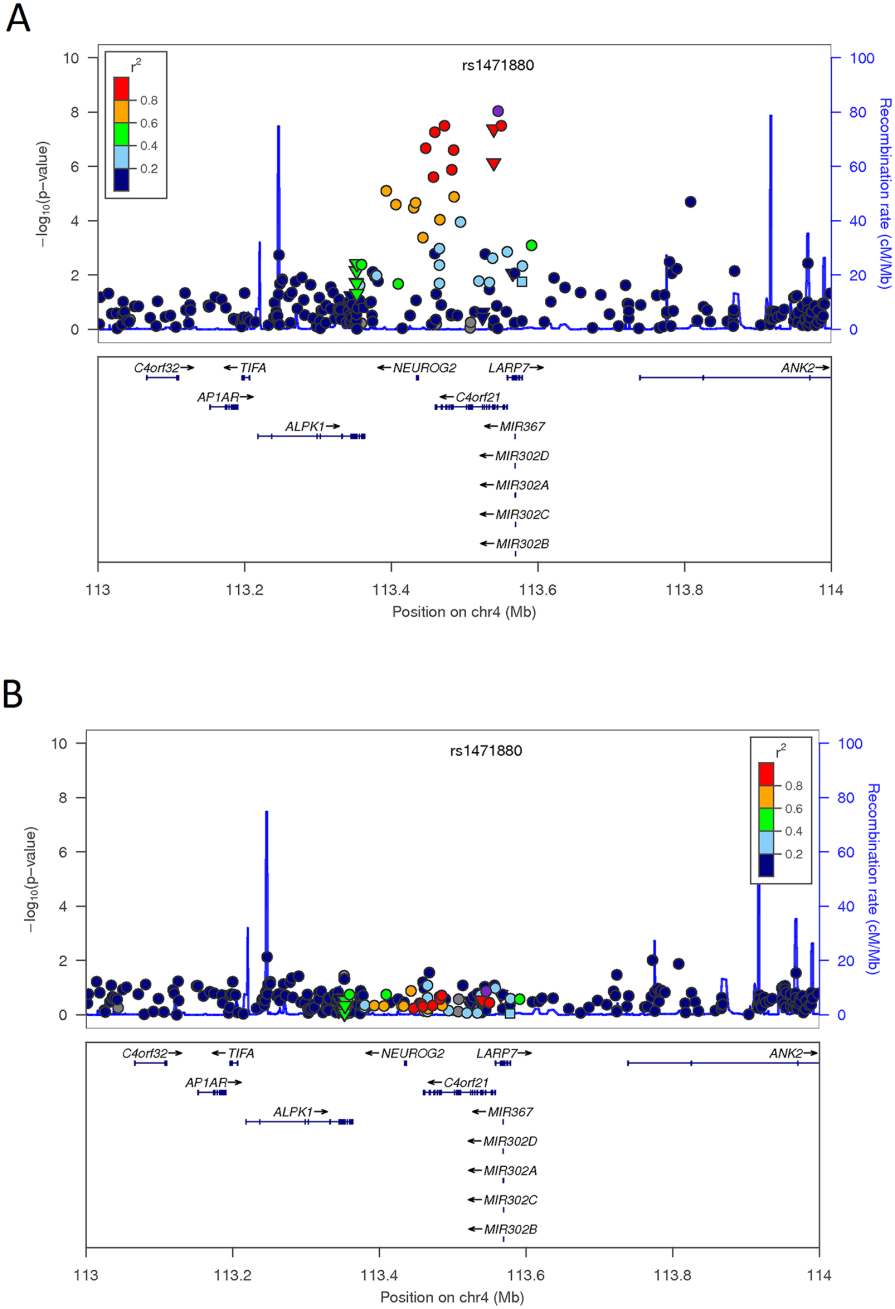
Regional plot of *ZGRF1* (*C4orf21*) for association with waist-hip ratio. (A). Analysis results in IRASFS for SNPs from genome-wide and exome chip datasets; (B). Conditioned on the most significant variant (rs1471880).–log_10_(p-values) under the best fit model are indicated on the left-hand Y axis. Association analyses were computed with adjustment for age, gender, recruitment center, and admixture estimates with SNP rs1471880 as an additional covariate in panel B. The recombination rates are indicated on the right-hand Y axis based on HapMap. The color of each SNP annotates its correlation (r^2^) with the index SNP and was taken from the 1000 Genomes AMR population. A circle denotes intronic and intergenic SNPs, a triangle denotes a missense SNP, and a square denotes a SNP in the untranslated region (UTR).

Overall, three intronic variants and one missense SNP in *ZGRF1* were identified above genome-wide significance for WHR (Table 2). The missense mutation (rs7696816) marks an asparagine to serine amino acid change with a benign effect predicted by PolyPhen [46]. The specific function of this gene remains unclear. The overall expression of *ZGRF1* in the human body is relatively low with the exception in brain and testis [47]. Direct replication of the *ZGRF1* signals was performed across six cohorts and the strongest signal from meta-analysis was rs1471880, P_DOM_ = 8.38x10^-6^ (Table 3). A consistent direction of effect was observed across the five larger cohorts (n_max_ = 3645) However, the statistical significance decreased (S7 Fig). Examination of this region in the GIANT (Genetic Investigation of Anthropometric Traits) Consortium for BMI and class 1 obesity (BMI>30) failed to reveal significant signals of association at the *ZGRF1* locus (P>0.01; S8 Fig) [15, 48]. Interestingly, previous studies have identified *ALPK1* (rs4833407), 100kb proximal to *ZGRF1*, to be associated with obesity in European populations [49]. However, the two SNPs in *ALPK1* and *ZGRF1* were poorly correlated in both CEU and IRASFS Hispanic Americans (r^2^ = 0.005 and 0.013, respectively). In IRASFS, most association signals centered around the *ZGRF1* locus with a few in *NEUROG2* and very weak signals in *ALPK1* (Fig 2). *NEUROG2* is a proneural protein neurogenin and has been shown to control cortical neuron migration through the regulation of small GTP-binding protein Rnd2 [50] and no direct link with adiposity has been established. Conditional analysis of this region with rs1471880 as a covariate abolished all association signals in *ZGRF1* as well as the signals in nearby *NEUROG2* without changes in *ALPK1* (Fig 2).

**Fig 3:**
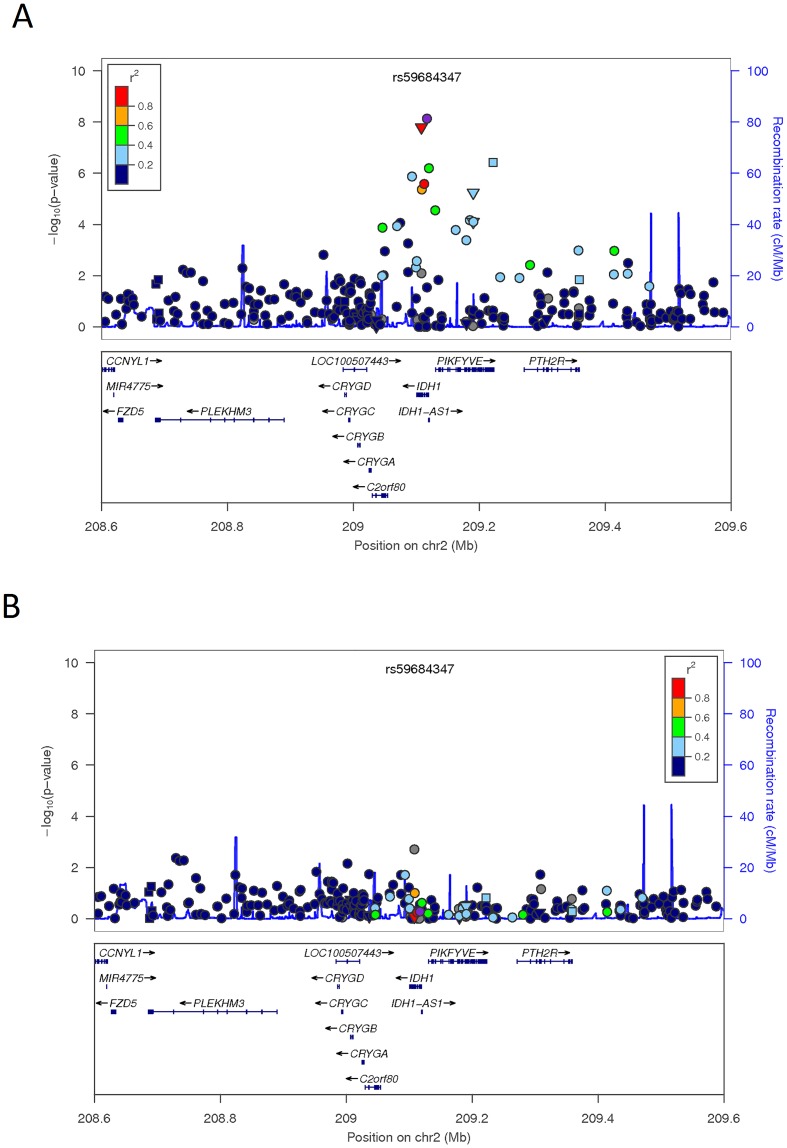
Regional plot of *IDH1* for association with waist circumference. (A). Analysis results in IRASFS for SNPs from genome-wide and exome chip datasets as well as de novo genotyping of the region; (B). Conditioned on rs34218846.–log_10_(p-values) under the best fit model are indicated on the left-hand Y axis. Association analyses were computed with adjustments for age, gender, recruiting center, and admixtures with SNP rs34218846 as an additional covariate in panel B. The recombination rates are indicated on the right-hand Y axis based on HapMap. The color of each SNP annotates its correlation (r^2^) with the index SNP and was taken from the 1000 Genomes AMR population. A circle denotes intronic and intergenic SNPs, a triangle denotes a missense SNP, and a square denotes a SNP in the untranslated region (UTR).

*IDH1* encodes cytosolic NADP+ dependent isocitrate dehydrogenase (IDPc) which has been proposed as a key enzyme for supplying cytosolic NADPH [51]. The most significant association signal observed was SNP rs34218846 (MAF = 0.068; P_DOM_ = 1.62x10^-8^) encoding a missense mutation from valine to isoleucine in exon 6 and was predicted as “probably damaging” by PolyPhen [46]. This mutation is located at the subunit dimerization interface, suggesting a potential regulatory role in gene function (S9 Fig). Previous genetic studies have suggested a strong correlation between *IDH1* mutations and cancer [52]. A biological link between *IDH1* and adiposity has been postulated using cell models. Specifically, stable transfection of *IDH1* cDNA positively correlated with adipogenesis of 3T3-L1 cells whereas decreased IDPc expression using an antisense IDPc vector retarded 3T3-L1 adipogenesis [53]. A more recent study reported knockdown of IDPc expression by RNA interference (RNAi) which inhibited adipocyte differentiation and lipogenesis in 3T3-L1 preadipocytes. In addition, in diet-induced obese mice transduced with IDPc short-hairpin RNA, a loss of body weight and reduction of triglyceride levels were observed [51]. The evaluation of serum triglyceride levels in IRASFS revealed carriers of rs34218846 T allele (adiposity protective allele) had a 20mg/dL decrease in triglyceride levels compared to non-carriers (P_DOM_ = 7.79x10^-3^). Taken together, *IDH1* appears to play an important role in fat metabolism. SNP rs34218846 was not directly genotyped among the replication cohorts. Therefore, two proxy SNPs, rs6435435 (P_DOM_ = 1.73x10^-6^ for BMI, r^2^ = 0.91 with rs34218846) and rs6734788 (P_ADD_ = 7.33x10^-7^ for WAIST, r^2^ = 0.37 with rs34218846), were selected for meta-analysis. However, these proxies failed to replicate (rs6435435 P_DOM_ = 6.74x10^-5^ for WAIST and rs6734788 P_ADD_ = 9.02x10^-5^ for WHR). Lack of association was similarly observed in IRAS (n = 184) with direct genotyping of rs34218846 (P = 0.45; S4 Table), which could be attributed reduced power given the small sample size. To search for additional putatively causal variants in *IDH1*, we conducted de novo genotyping in IRASFS which revealed an intronic SNP (rs59684347) showing stronger evidence of association (P_ADD_ = 7.42x10^-9^; WAIST) (Fig 3). However, rs34218846 and rs59684347 were highly correlated (r^2^ = 1.00) and all evidence of association in the region was abolished after inclusion of rs34218846 as a covariate in the analysis (Fig 3). Overall, *IDH1* represents a promising locus with evidence of association to adiposity-related traits, especially waist circumference. Notably, larger cohorts from European-derived populations in the GIANT Consortium have identified BMI associated signals in *CRYGD* (rs10932241), which is 100kb proximal to *IDH1*. However, there was no signal of association for the *IDH1* locus in GIANT and rs10932241 was poorly correlated with rs34218846 (r^2^ = 0.057) and only nominally associated with BMI (p-value = 0.057; S10 Fig) in IRASFS despite a similar minor allele frequency observed in European populations (MAF = 5.31%) [15, 48].

In summary, although encouraging results have been revealed, there are several study limitations. Like most minority studies, sample size largely limited the power, especially for rare variants assessed on the exome chip. In addition, the utility of the Illumina HumanExome Array in Hispanic Americans is not optimal as only 81,559 out of 242,901 SNPs on the array were polymorphic, likely attributable to a design based on findings in Caucasians and African Americans. The application of Illumina OmniExpress BeadChip has similar concerns: the SNPs on the chip may not tag the LD structure as well in Hispanic Americans. Another issue is the lack of replication signals: all signals fell below the significance threshold after meta-analysis. There are several possible reasons: first, the replication cohorts were limited to directly genotyped GWAS variants and we were unable to replicate signals from the exome chip among all cohorts. Second, some replication cohorts did not have CT and DEXA measures for replication, necessitating the use of surrogate phenotypes. Third, while all cohorts were of Hispanic ancestry, different ascertainment criteria were used. For example, BetaGene recruited participants at high risk of gestational diabetes while HTN-IR recruited participants at high risk of hypertension. This differs from IRASFS which is a population-based study recruited based on large family size. Additionally, the sample sizes for IRAS, TRIPOD, and NIDDM-Athero were relatively small. This may explain why the more significant associations, e.g. rs1471880 demonstrated an opposite direction of effect in TRIPOD (n = 125) and NIDDM-Athero (n = 179) (S7 Fig). Another concern is the large effects of *IDH1* (2.1%) and *ZGRF1* (2.7%) in this study are weak from previous European population studies (S8 and S10 Figs). One explanation is the potential for ethnic-specific variants or that the signals are the result of gene-environment effects. It is also possible that the signals observed are not causal and they were detected due to a long range LD with other loci.

Until now, VIVA LA FAMILIA was the only cohort with published genome-wide significant obesity-related signals specific to the Hispanic population [23]. Further evaluation of the obesity-related loci from VIVA LA FAMILIA in IRASFS revealed nominal association for rs2823615 (P_DOM_ = 7.86x10^-3^ with SAT), an intronic SNP in the Family with Sequence Similarity 222 Member A gene (*FAM222A*). This SNP has been shown to be associated with increased respiratory quotient in VIVA LA FAMILIA and increased SAT in IRASFS.

In summary, we computed a combined study of genome-wide and exome chip arrays in the IRASFS Hispanic-American population. Six obesity related traits were analyzed for association. *ZGRF1* and *IDH1* attained genome-wide significance in IRASFS and replication of significant signals was evaluated in six additional Hispanic cohorts (n_max_ = 4155). Meta-analysis suggested decreased levels of significance (*ZGRF1* rs1471880, P_DOM_ = 8.38x10^-6^; *IDH1* rs6435435, P_DOM_ = 6.74x10^-5^). These results highlight the importance of GWAS and exome chip research in minority populations where an increased prevalence of adiposity-related diseases may be associated with a differential genetic architecture than in European-derived populations.

## Supporting Information

S1 Fig QQ plots of the association results from IRASFS for BMI. (PNG)

S2 Fig QQ plots of the association results from IRASFS for WAIST. (PNG)

S3 Fig QQ plots of the association results from IRASFS for WHR. (PNG)

S4 Fig QQ plots of the association results from IRASFS for SAT. (PNG)

S5 Fig QQ plots of the association results from IRASFS for VAT. (PNG)

S6 Fig QQ plots of the association results from IRASFS for PBF. (PNG)

S7 Fig Forest plot of the effect for *ZGRF1* SNP rs1471880 in all 7 cohorts for WAIST based on the A allele under the dominant genetic model. For each study, data presented represent the log(WAIST) beta coefficient indexed to the standard error. Bars mark the 95% confidence intervals.
(TIF)

S8 Fig *ZGRF1* (*C4orf21*) regional signals from the GIANT consortium. **A**. BMI; **B**. Class 1 obesity. -log_10_(p-values) are indicated on the left-hand Y axis. The recombination rates are indicated on the right-hand Y axis based on HapMap.
(TIF)

S9 Fig Protein structure and regional view of human cytosolic NADP(+)-dependent isocitrate dehydrogenase. The two dimers are colored in white and yellow. **A.** Amino acid 178 (rs34218846 valine to isoleucine) is indicated by an arrow. **B.** A regional view of amino acid 178 with the side chain colored in red.
(TIF)

S10 Fig *IDH1* regional signals from the GIANT consortium. **A**. BMI; **B**. Class 1 obesity.–log_10_(p-values) are indicated on the left-hand Y axis. The recombination rates are indicated on the right-hand Y axis under 1000 Genomes CEU.
(TIF)

S1 Table Summary of de novo genotyping in IRASFS. (XLSX)

S2 Table Meta-analysis results for significant signals of association (5.00x10^-6^) from IRASFS. (XLSX)

S3 Table Meta-analysis of replication cohorts only for significant signals of association (5.00x10^-6^) from IRASFS. (XLSX)

S4 Table Summary of de novo genotyping in IRAS. (XLSX)

S5 Table Summary of previously published genome-wide significant (P<5.00x10^-8^) adiposity signals in IRASFS. (XLSX)

S6 Table The number of significant signals for each trait and P-values for sign tests. (XLSX)

S1 Text Supplemental material-detailed descriptions of genotyping, quality control, and replication cohorts. (DOCX)
