# Supplementary figures and images for: A Comprehensive Analysis of Common and Rare Variants to Identify Adiposity Loci in Hispanic Americans: The IRAS Family Study (IRASFS)
Source: PLoS One. 2015 Nov 24;10(11):e0134649. doi: 10.1371/journal.pone.0134649 (PMC4658008; doi:10.1371/journal.pone.0134649)

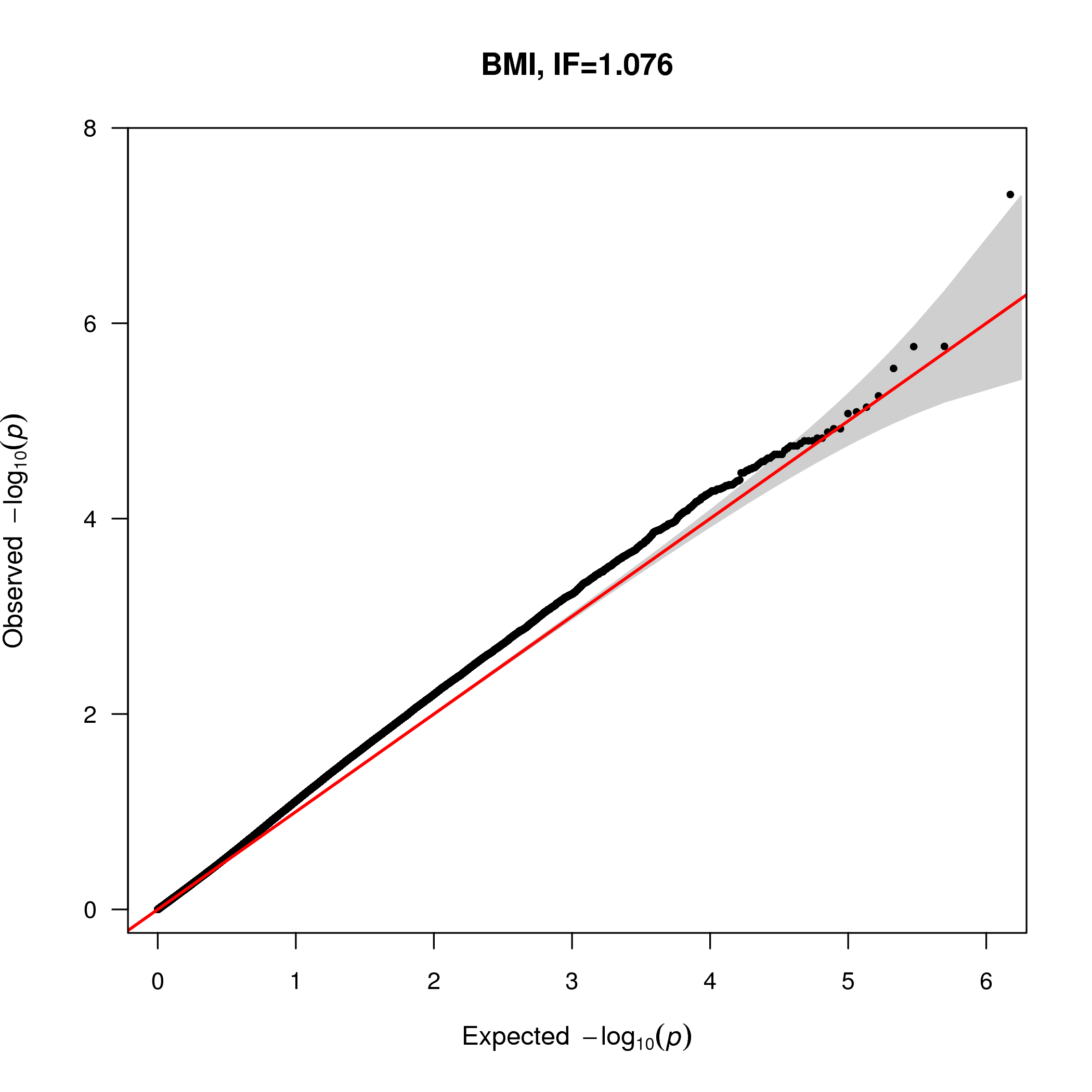

Supplement: S1 Fig — (PNG) [file pone.0134649.s001.png]

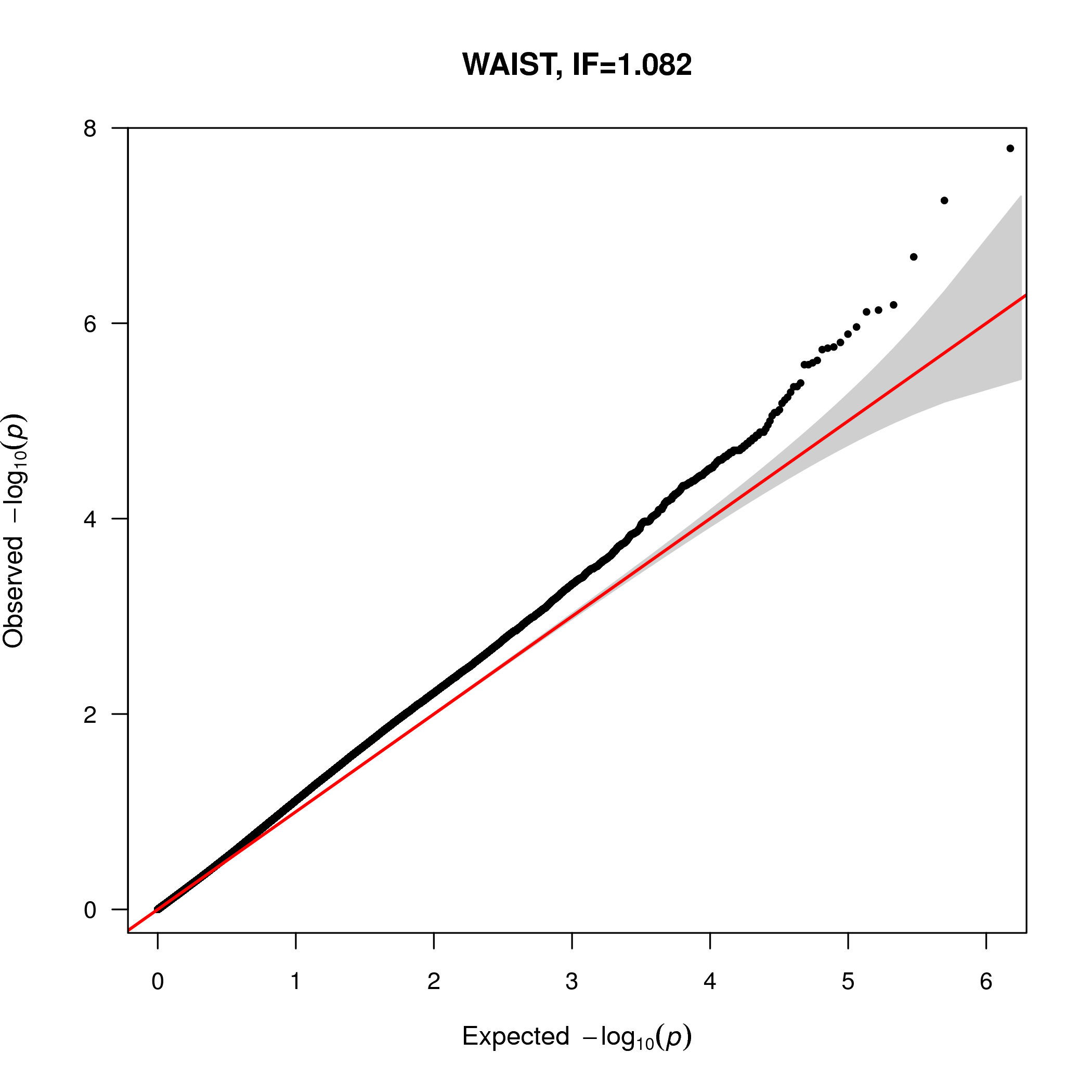

Supplement: S2 Fig — (PNG) [file pone.0134649.s002.png]

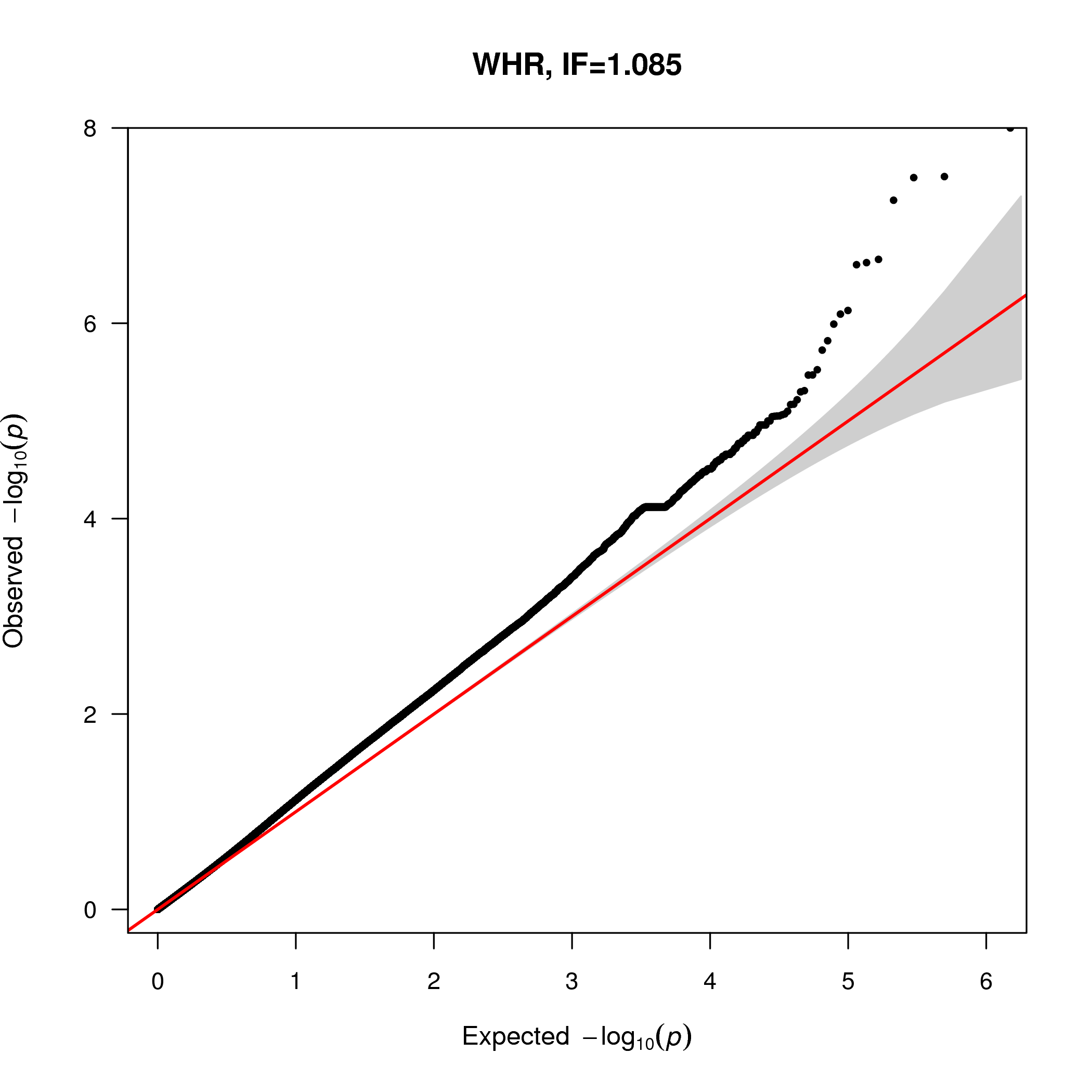

Supplement: S3 Fig — (PNG) [file pone.0134649.s003.png]

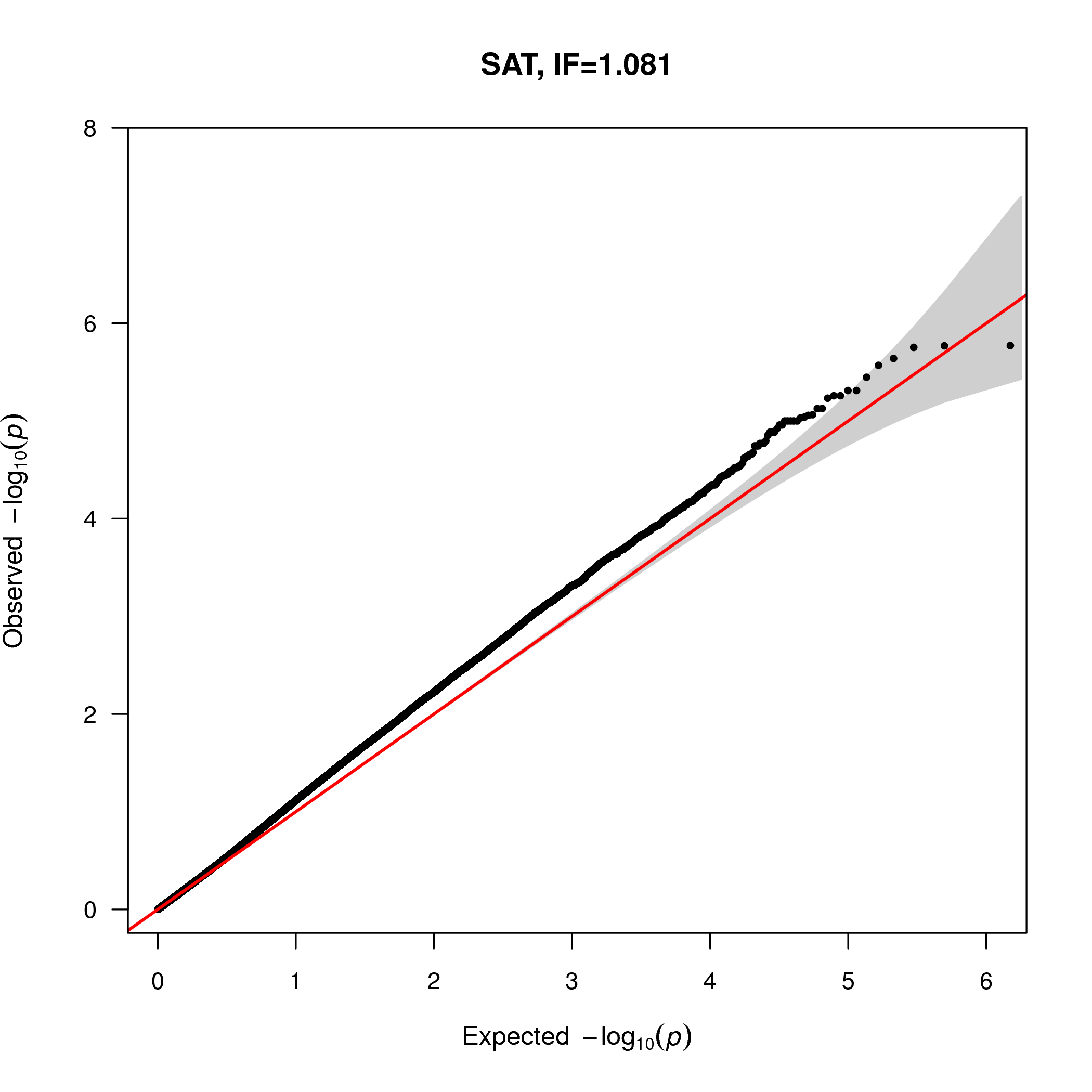

Supplement: S4 Fig — (PNG) [file pone.0134649.s004.png]

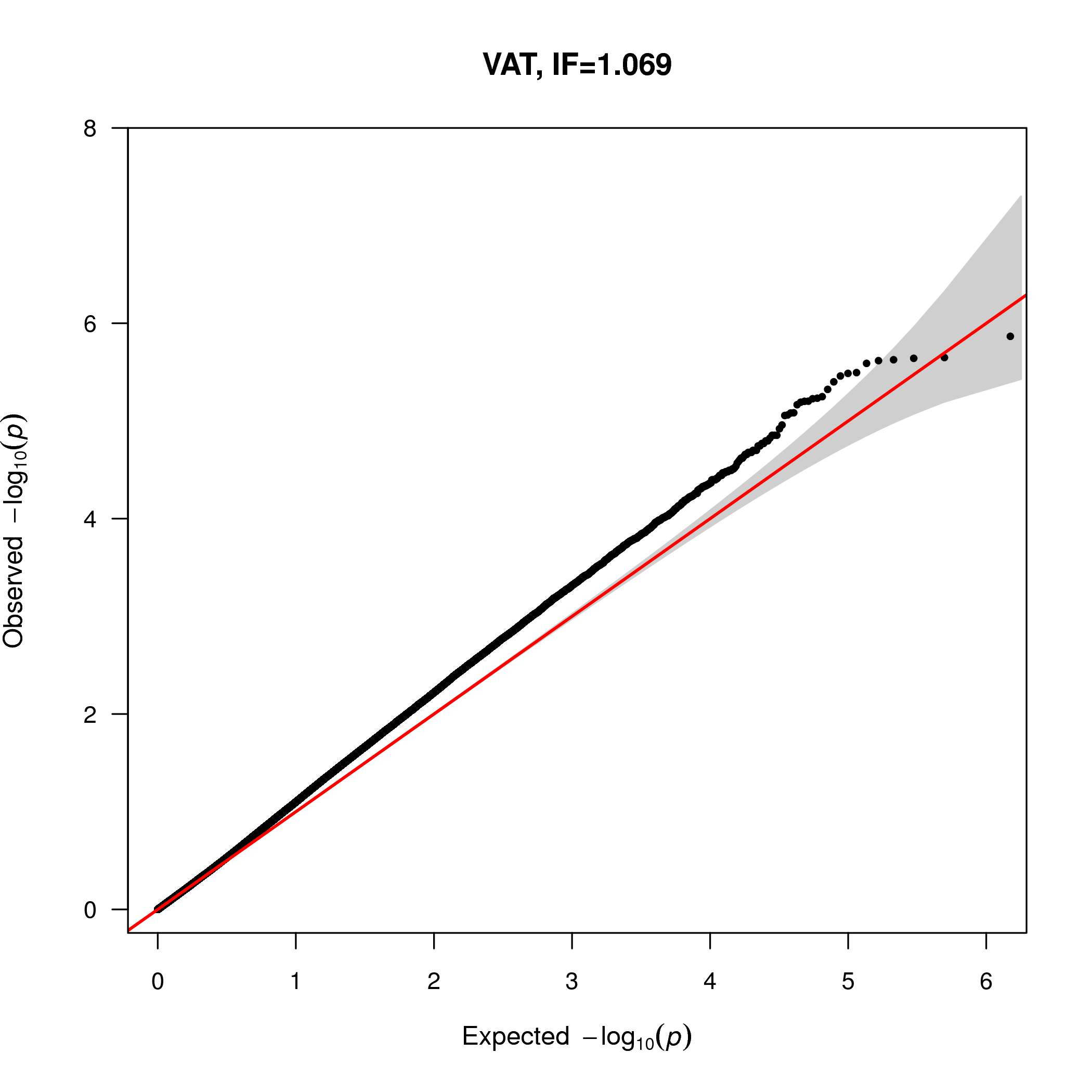

Supplement: S5 Fig — (PNG) [file pone.0134649.s005.png]

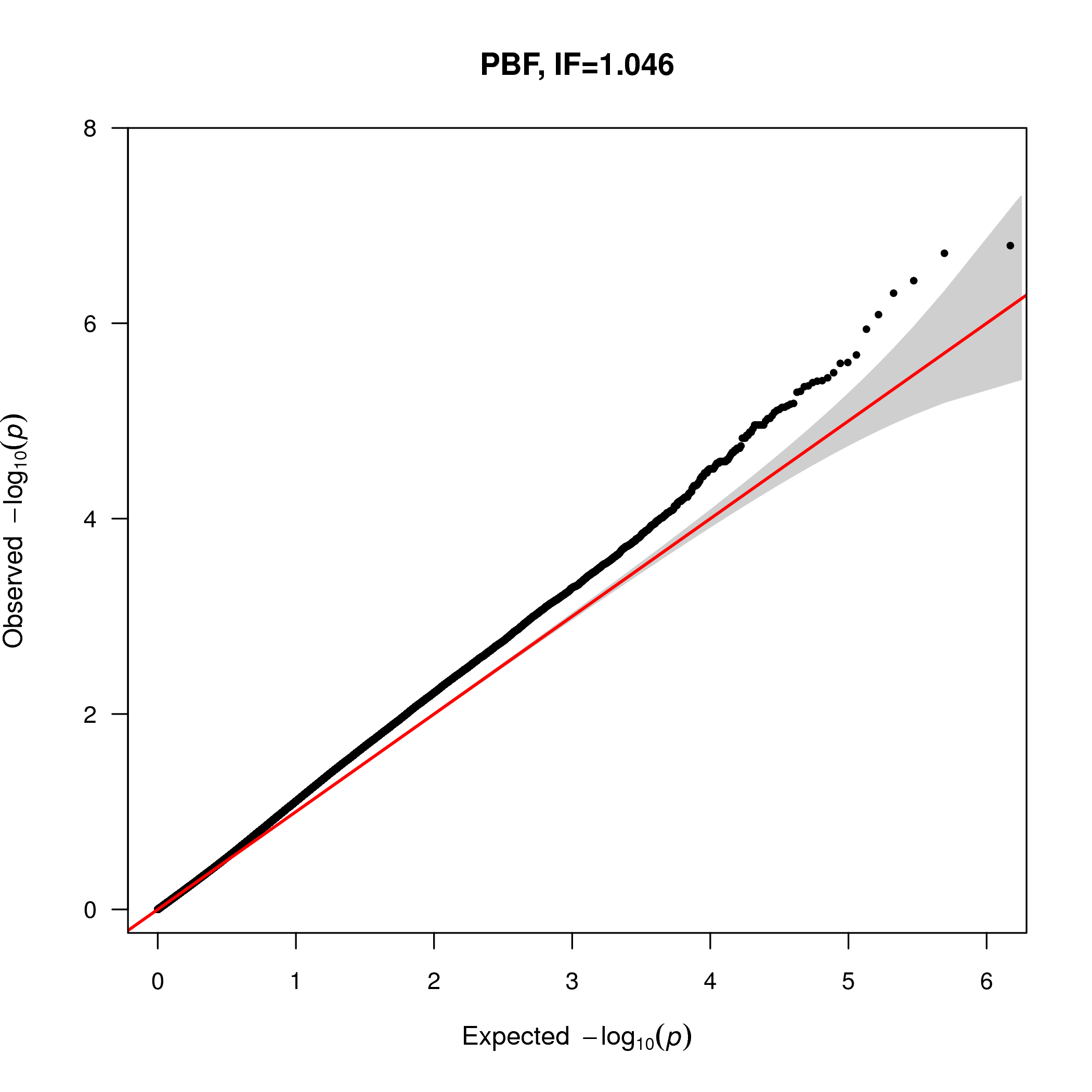

Supplement: S6 Fig — (PNG) [file pone.0134649.s006.png]

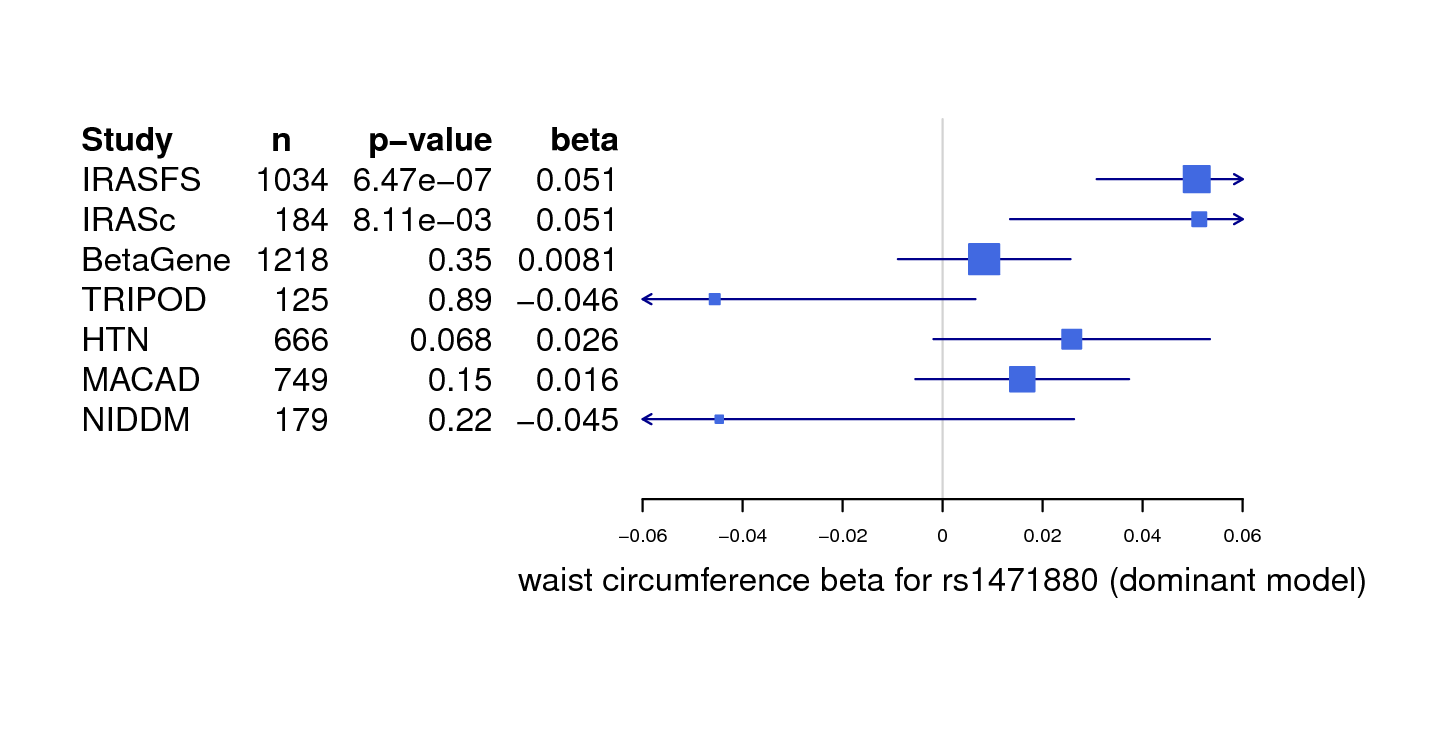

Supplement: S7 Fig — For each study, data presented represent the log(WAIST) beta coefficient indexed to the standard error. Bars mark the 95% confidence intervals. (TIF) [file pone.0134649.s007.tif]

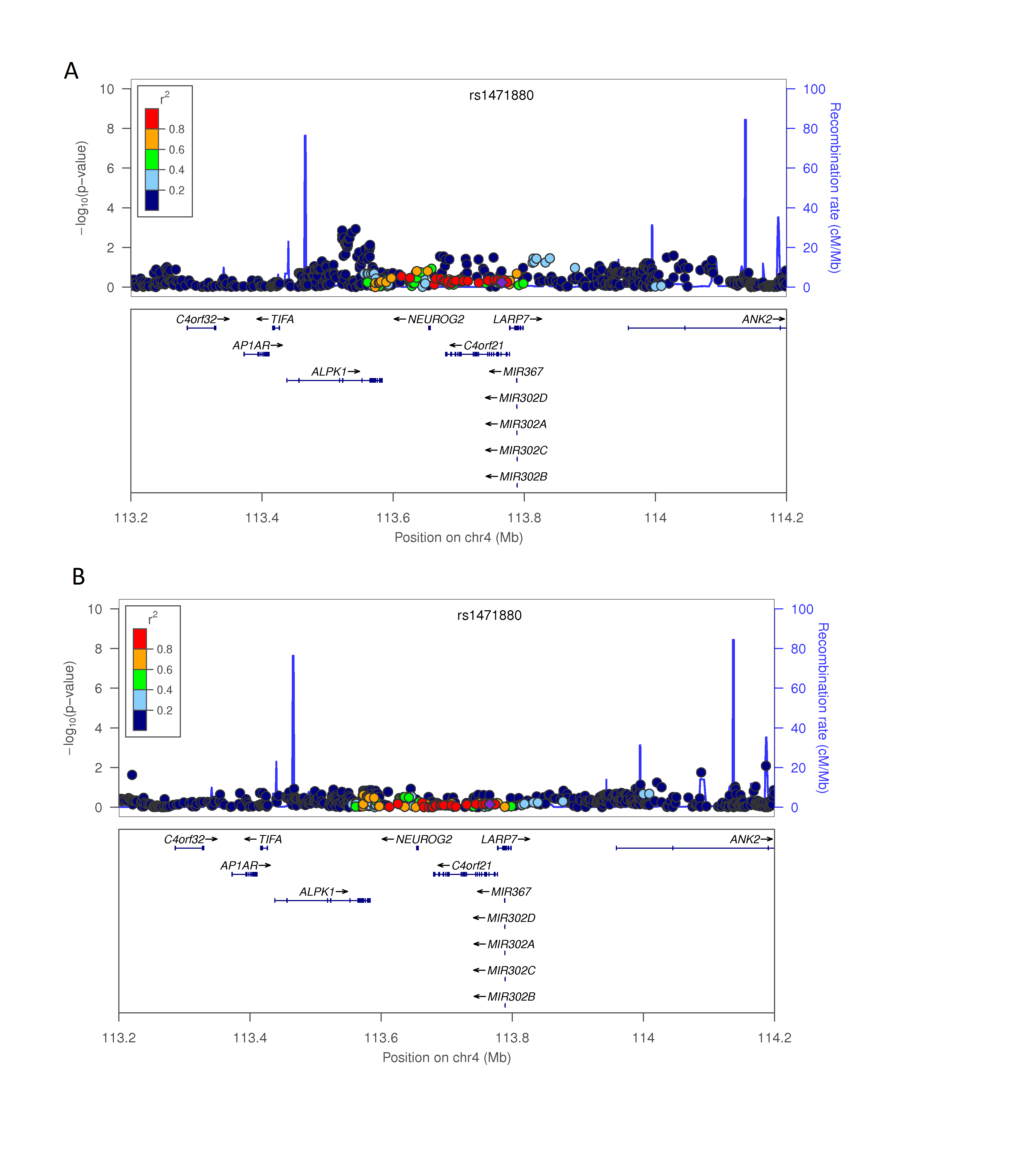

Supplement: S8 Fig — A. BMI; B. Class 1 obesity. -log10(p-values) are indicated on the left-hand Y axis. The recombination rates are indicated on the right-hand Y axis based on HapMap. (TIF) [file pone.0134649.s008.tif]

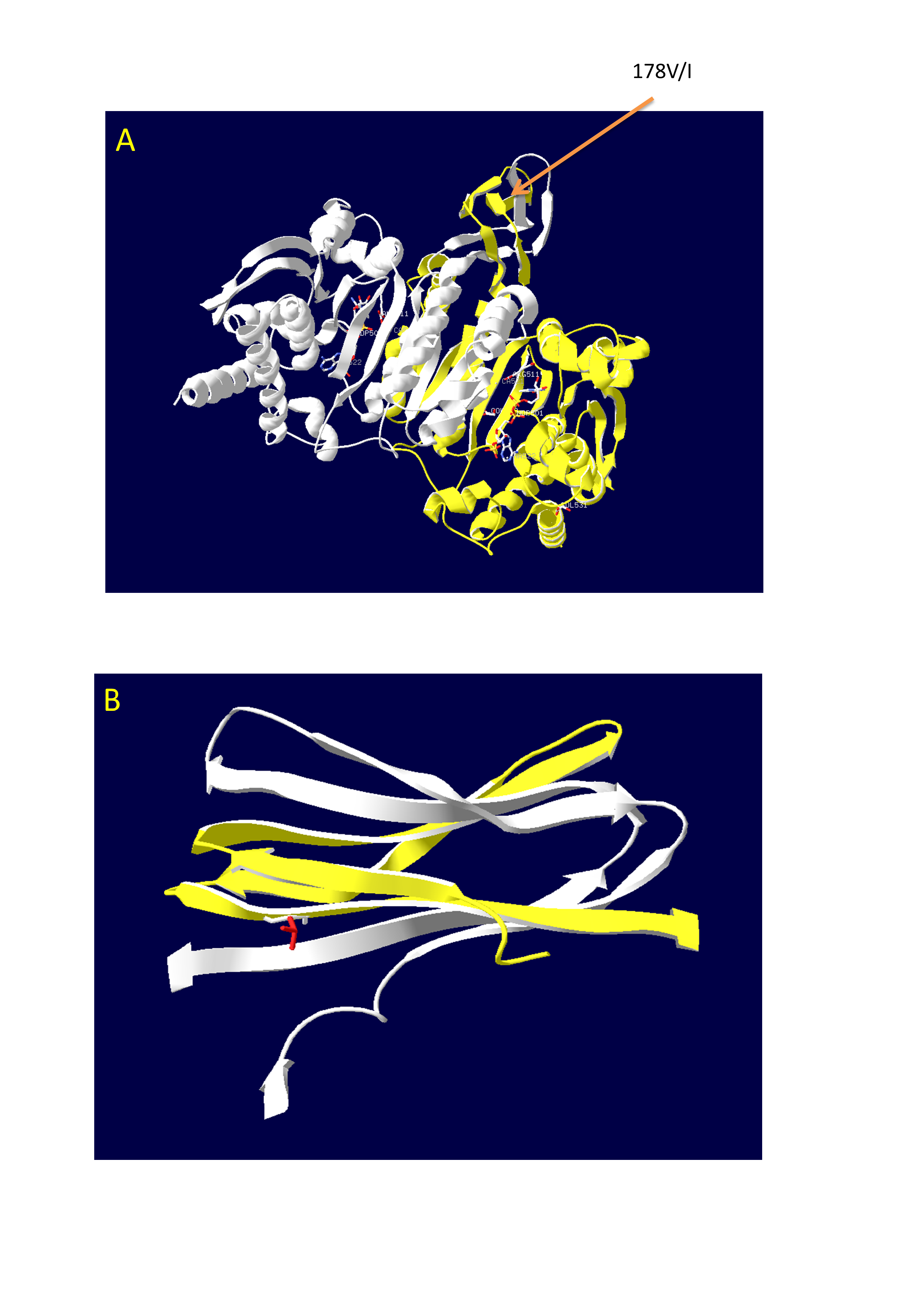

Supplement: S9 Fig — The two dimers are colored in white and yellow. A. Amino acid 178 (rs34218846 valine to isoleucine) is indicated by an arrow. B. A regional view of amino acid 178 with the side chain colored in red. (TIF) [file pone.0134649.s009.tif]

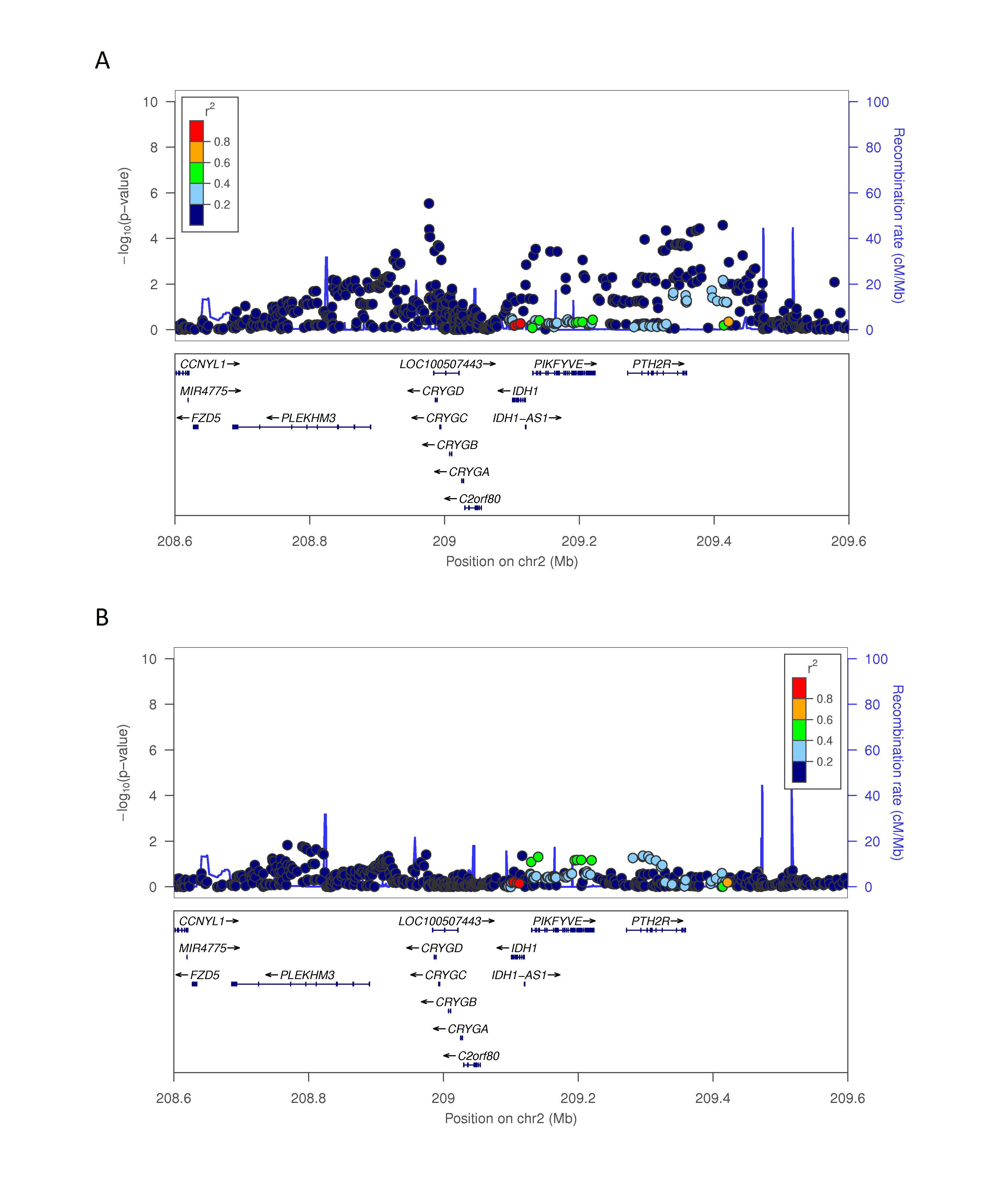

Supplement: S10 Fig — A. BMI; B. Class 1 obesity.–log10(p-values) are indicated on the left-hand Y axis. The recombination rates are indicated on the right-hand Y axis under 1000 Genomes CEU. (TIF) [file pone.0134649.s010.tif]
