# Supplementary material for: A Comprehensive Analysis of Common and Rare Variants to Identify Adiposity Loci in Hispanic Americans: The IRAS Family Study (IRASFS)
Source: PLoS One. 2015 Nov 24;10(11):e0134649. doi: 10.1371/journal.pone.0134649 (PMC4658008; doi:10.1371/journal.pone.0134649)
Supplement: S1 Text — (DOCX) [file pone.0134649.s017.docx]

**Supplemental material**

**Genotyping and Quality Control**

*GWAS*

Genomic DNA was isolated using PUREGENE DNA isolation kits (Gentra Inc., Minneapolis, MN, USA). DNA was quantitated using a NanoDrop Spectrophotometer (Thermo Scientific; Wilmington, DE, USA) and concentrations were standardized to 50ng/µL for genotyping. Genotyping of the IRASFS cohort was supported through the Genetics Underlying Diabetes in Hispanics (GUARDIAN) Consortium ([4](#_ENREF_4)) which aims to identify the genetic architecture of quantitative measures of insulin resistance and as such, excluded individuals diagnosed with T2D. Genotyping was performed in the genotyping laboratory of the Common Disease Genetic Group formerly located at Cedars-Sinai Medical Center, Los Angeles, CA, USA now located at the Los Angeles Biomedical Research Institute, Los Angeles, CA, USA. Genotyping was attempted for 1039 Hispanic Americans plus 13 quality control (QC) duplicates using the Illumina OmniExpress Array (Illumina Inc.; San Diego, CA, USA; n=730,525 markers) with an additional 14 external controls included to verify reproducibility across genotyping runs. Concordance across external controls was greater than 99.99%. Normalization of raw image intensity data, genotype clustering, and individual sample genotype calls were made using the Illumina GenomeStudio software. Samples were repeated if call rates were less than 98%. The final average call rate per sample was greater than 99.6% with reproducibility of within plate duplicates greater than 99.99%. SNPs with a p10GC score less than 0.75, call rates less than 95%, and excess heterozygosity were excluded. QC duplicates and gender checks were analyzed using PLINK ([5](#_ENREF_5)) and SNP fingerprinting analysis was computed using pre-existing genotype data from 54 SNPs with a MAF greater than 0.1. This resulted in a final analysis dataset of 717,400 SNPs across 1034 individuals.

*Exome Chip*

Exome chip genotyping was carried out on the Illumina HumanExome Array v1.0 (n=560) and v1.1 (n=864) (Illumina Inc.; San Diego, CA, USA) in the Center for Genomics and Personalized Medicine Research at Wake Forest School of Medicine, Winston-Salem, NC, USA. Genotype calling was performed using Illumina GenomeStudio software. Quality control procedures were unified across both array versions. Samples with a call rate less than 99% were repeated (*n*=1 for v1.0). SNP level exclusions were made for SNPs with poor cluster separation (less than 0.35; n=282 for v1.0 and n=1019 for v1.1) and low call rates (less than 99%; n=1147 for v1.0 and n=1250 for v1.1). Manual review of gender calling using data from the X and Y chromosomes revealed five samples discordant with documented genders which were subsequently dropped from the analysis. Merged genotypes from v1.0 and v1.1 were analyzed with PedCheck ([6](#_ENREF_6)) to identify Mendelian inconsistencies resulting in the removal of five samples due to high error rates. Additional manual review of SNPs with high error rates (>5 Mendelian inconsistencies) resulted in zeroing of 97 SNPs. The final dataset consisted of 81,559 autosomal SNPs from 1414 IRASFS Hispanic Americans which included 380 individuals excluded from GWAS for the absence of glucose homeostasis measures including 161 diabetic individuals.

**List of obesity-related phenotypes from GWAS Catalog**

Adiposity; Body mass (lean); Body mass index; Obesity; Obesity (extreme); Obesity-related traits; Adiposity in newborns; Subcutaneous adipose tissue; Visceral adipose tissue adjusted for BMI; Visceral adipose tissue; Visceral adipose tissue/subcutaneous adipose tissue ratio; Waist circumference; Waist to hip ratio; Weight.

**Replication cohorts**

*Insulin Resistance Atherosclerosis Study (IRAS)*

IRAS was the first epidemiologic study designed to assess the relationship between insulin resistance and prevalent CVD in a large multiethnic cohort. Hispanic Americans included in this cohort were recruited from San Antonio, TX and San Luis Valley, CO (n=548) representing three stages of glucose tolerance: normal glucose tolerance (n=241), impaired glucose tolerance (n=123), and T2D (n=184). Exclusion of subjects overlapping with IRASFS resulted in the evaluation of 184 individuals for replication. In silico replication was performed on GWAS data generated from the Illumina OmniExpress Array. Available phenotypes include Body Mass Index (BMI), Waist Circumference (WAIST), and Waist-Hip Ratio (WHR). A full description of the design and methods for IRAS has been published ([9](#_ENREF_9)). The IRAS protocol was approved by the local institutional review committees and all participants gave their informed consent.

*BetaGene*

BetaGene is a family-based study of obesity, insulin resistance, and beta-cell dysfunction in Hispanic Americans. Details of the recruitment strategy ([10](#_ENREF_10)) and clinical examination ([11-13](#_ENREF_11)) have been previously described. Individuals were identified from the patient populations at Los Angeles County/University of Southern California Medical Center, the Kaiser Permanente Southern California health plan membership, and obstetric/gynecologic clinics at local southern California hospitals. Participants were Hispanic Americans (both parents and ≥3 grandparents were Mexican or of Mexican descent) who were either 1) women diagnosed with gestational diabetes mellitus (GDM) within the previous 5 years, 2) siblings or cousins of those with a history of GDM, or 3) women with normal glucose concentrations during pregnancy in the past 5 years. In silico replication was performed through querying of data generated from the Illumina OmniExpress Array in 1218 individuals. Phenotypes available for replication include BMI, WAIST, WHR, and Percent Body Fat (PBF). Protocols for BetaGene were approved by the institutional review boards of participating institutions and all participants provided written informed consent.

*Troglitazone in Prevention of Diabetes (TRIPOD) Study*

The details about the TRIPOD Study have been previously described ([14](#_ENREF_14), [15](#_ENREF_15)). Brieﬂy, subjects were recruited from Los Angeles County Women’s and Children’s Hospital. Criteria for participation were Hispanic ethnicity, non-diabetic but had gestational diabetes in the previous 4 years, and a sum of ﬁve oral glucose tolerance test plasma glucose concentrations of at least 625 mg/dL (34.7 mmol/l), predicting a 70% risk of diabetes in the next 5 years. In silico replication was performed based on Illumina OmniExpress Array data of 125 individuals. Phenotypes available for replication include BMI, WAIST, and WHR. Protocols for TRIPOD were approved by the institutional review boards of participating institutions and all participants provided written informed consent.

*Hypertension-Insulin Resistance (HTN-IR) Family Study*

The HTN-IR Family Study was designed to examine the genetic basis of hypertension and insulin resistance ([16](#_ENREF_16), [17](#_ENREF_17)). Families were ascertained through hypertensive probands in the Los Angeles, CA area. In silico replication was performed based on Illumina OmniExpress Array data of 666 individuals. Phenotypes available for replication include BMI, WAIST, WHR. PBF is available for a reduced number of individuals (n=139). Written consent for participation was approved by the Institutional Review Board of the Los Angeles County – University of Southern California Medical Center.

*Mexican-American Coronary Artery Disease (MACAD) Study*

The UCLA/Cedars-Sinai MACAD Study was designed to examine the genetic basis of coronary artery disease (CAD) and insulin resistance. Families ascertained through a proband with documented CAD were recruited in the Los Angeles, CA area ([18-20](#_ENREF_18)). Replication data was provided for 749 individuals using the Illumina OmniExpress Array. Phenotypes available for replication include BMI, WAIST, WHR, and PBF. All studies were approved by Human Subjects Protection Institutional Review Boards at UCLA, Cedars-Sinai. All subjects gave informed consent prior to participation.

*NIDDM-Atherosclerosis (NIDDM-Athero) Study*

The NIDDM-Athero Study was designed as a family study to examine the genetic basis of subclinical atherosclerosis and diabetes. Family members of probands with T2D were recruited in the Los Angeles, CA area. A total of 179 individuals from 93 families were included for replication analysis using OmniExpress Array ([21](#_ENREF_21)). Phenotypes available for replication include BMI, WAIST, and WHR. The NIDDM-Athero protocol was approved by the local institutional review committees and all participants gave their informed consent.

1. Alexander DH, Novembre J, Lange K. Fast model-based estimation of ancestry in unrelated individuals. Genome Res 2009;19(9):1655-64.

2. Almasy L, Blangero J. Multipoint quantitative-trait linkage analysis in general pedigrees. Am J Hum Genet 1998;62(5):1198-211.

3. Norris JM, Langefeld CD, Scherzinger AL, Rich SS, Bookman E, Beck SR, et al. Quantitative trait loci for abdominal fat and BMI in Hispanic-Americans and African-Americans: the IRAS Family study. Int J Obes (Lond) 2005;29(1):67-77.

4. Goodarzi MO, Langefeld CD, Xiang AH, Chen YD, Guo X, Hanley AJ, et al. Insulin sensitivity and insulin clearance are heritable and have strong genetic correlation in Mexican Americans. Obesity (Silver Spring) 2014;22(4):1157-64.

5. Purcell S, Neale B, Todd-Brown K, Thomas L, Ferreira MA, Bender D, et al. PLINK: a tool set for whole-genome association and population-based linkage analyses. Am J Hum Genet 2007;81(3):559-75.

6. O'Connell JR, Weeks DE. PedCheck: a program for identification of genotype incompatibilities in linkage analysis. Am J Hum Genet 1998;63(1):259-66.

7. Hindorff LA MJEBI, Morales J (European Bioinformatics Institute), Junkins HA, Hall PN, Klemm AK, and Manolio TA. A Catalog of Published Genome-Wide Association Studies. Available at: [www.genome.gov/gwastudies](http://www.genome.gov/gwastudies). Accessed 10/16/2013.

8. Howie BN, Donnelly P, Marchini J. A flexible and accurate genotype imputation method for the next generation of genome-wide association studies. PLoS Genet 2009;5(6):e1000529.

9. Wagenknecht LE, Mayer EJ, Rewers M, Haffner S, Selby J, Borok GM, et al. The insulin resistance atherosclerosis study (IRAS) objectives, design, and recruitment results. Ann Epidemiol 1995;5(6):464-72.

10. Watanabe RM, Allayee H, Xiang AH, Trigo E, Hartiala J, Lawrence JM, et al. Transcription factor 7-like 2 (TCF7L2) is associated with gestational diabetes mellitus and interacts with adiposity to alter insulin secretion in Mexican Americans. Diabetes 2007;56(5):1481-5.

11. Black MH, Fingerlin TE, Allayee H, Zhang W, Xiang AH, Trigo E, et al. Evidence of interaction between PPARG2 and HNF4A contributing to variation in insulin sensitivity in Mexican Americans. Diabetes 2008;57(4):1048-56.

12. Li X, Allayee H, Xiang AH, Trigo E, Hartiala J, Lawrence JM, et al. Variation in IGF2BP2 interacts with adiposity to alter insulin sensitivity in Mexican Americans. Obesity (Silver Spring) 2009;17(4):729-36.

13. Shu YH, Hartiala J, Xiang AH, Trigo E, Lawrence JM, Allayee H, et al. Evidence for sex-specific associations between variation in acid phosphatase locus 1 (ACP1) and insulin sensitivity in Mexican-Americans. J Clin Endocrinol Metab 2009;94(10):4094-102.

14. Buchanan TA, Xiang AH, Peters RK, Kjos SL, Marroquin A, Goico J, et al. Preservation of pancreatic beta-cell function and prevention of type 2 diabetes by pharmacological treatment of insulin resistance in high-risk hispanic women. Diabetes 2002;51(9):2796-803.

15. Buchanan TA, Xiang AH, Peters RK, Kjos SL, Berkowitz K, Marroquin A, et al. Response of pancreatic beta-cells to improved insulin sensitivity in women at high risk for type 2 diabetes. Diabetes 2000;49(5):782-8.

16. Xiang AH, Azen SP, Raffel LJ, Tan S, Cheng LS, Diaz J, et al. Evidence for joint genetic control of insulin sensitivity and systolic blood pressure in hispanic families with a hypertensive proband. Circulation 2001;103(1):78-83.

17. Cheng LS, Davis RC, Raffel LJ, Xiang AH, Wang N, Quinones M, et al. Coincident linkage of fasting plasma insulin and blood pressure to chromosome 7q in hypertensive hispanic families. Circulation 2001;104(11):1255-60.

18. Goodarzi MO, Guo X, Taylor KD, Quinones MJ, Samayoa C, Yang H, et al. Determination and use of haplotypes: ethnic comparison and association of the lipoprotein lipase gene and coronary artery disease in Mexican-Americans. Genet Med 2003;5(4):322-7.

19. Goodarzi MO, Guo X, Taylor KD, Quinones MJ, Saad MF, Yang H, et al. Lipoprotein lipase is a gene for insulin resistance in Mexican Americans. Diabetes 2004;53(1):214-20.

20. Goodarzi MO, Taylor KD, Guo X, Quinones MJ, Cui J, Li X, et al. Variation in the gene for muscle-specific AMP deaminase is associated with insulin clearance, a highly heritable trait. Diabetes 2005;54(4):1222-7.

21. Wang Y-P, Kandeel F, Taylor KD, Hernandez D, Saad MF, Nadler JL, et al. Insulin and blood pressure are linked to the LDL receptor-related protein locus on chromosome 12q (Abstract). Diabetes 2000;49(Supp 1):A204.
